# FTD/ALS-associated poly(GR) protein impairs the Notch pathway and is recruited by poly(GA) into cytoplasmic inclusions

**DOI:** 10.1007/s00401-015-1448-6

**Published:** 2015-06-02

**Authors:** Dejun Yang, Abbas Abdallah, Zhaodong Li, Yubing Lu, Sandra Almeida, Fen-Biao Gao

**Affiliations:** Department of Neurology, University of Massachusetts Medical School, 368 Plantation Street, Worcester, MA 01605 USA; Department of Pediatric Oncology, Dana-Farber Cancer Institute, Harvard Medical School, Boston, MA 02216 USA

**Keywords:** ALS, DPR, *Drosophila*, FTD, Inclusion, Motor neuron, Notch, Poly(GA), Poly(GR), Poly(PR), RAN translation

## Abstract

*C9ORF72* repeat expansion is the most common genetic mutation in frontotemporal dementia (FTD) and amyotrophic lateral sclerosis (ALS). Abnormal dipeptide repeat proteins (DPRs) generated from repeat-associated non-AUG (RAN) translation of repeat-containing RNAs are thought to be pathogenic; however, the mechanisms are unknown. Here we report that (GR)_80_ and (PR)_80_ are toxic in neuronal and non-neuronal cells in *Drosophila*. In contrast to reported shorter poly(GR) forms, (GR)_80_ is mostly localized throughout the cytosol without detectable accumulation in the nucleolus, accompanied by suppression of Notch signaling and cell loss in the wing. Some Notch target genes are also downregulated in brains and iPSC-derived cortical neurons of *C9ORF72* patients. Increased Notch expression largely suppressed (GR)_80_-induced cell loss in the wing. When co-expressed in *Drosophila,* HeLa cells, or human neurons, (GA)_80_ recruited (GR)_80_ into cytoplasmic inclusions, partially decreasing the toxicity of (GR)_80_ and restoring Notch signaling in *Drosophila*. Thus, different DPRs have opposing roles in cell loss and we identify the Notch pathway as one of the receptor signaling pathways that might be compromised in C9ORF72 FTD/ALS.

**Electronic supplementary material:**

The online version of this article (doi:10.1007/s00401-015-1448-6) contains supplementary material, which is available to authorized users.

## Introduction

Frontotemporal dementia (FTD) and amyotrophic lateral sclerosis (ALS) share many pathological, genetic, and molecular features [23, 25, 32]. Indeed, in both disorders, GGGGCC (G_4_C_2_) repeat expansion in the noncoding region of *C9ORF72* is the most common genetic mutation [10, 16, 31]. G_4_C_2_ sense and antisense transcripts form RNA foci in patient brains, fibroblasts, induced pluripotent stem cells (iPSCs), and iPSC-derived neurons [1, 10, 13, 15, 22, 27, 34, 42], which may contribute to disease pathogenesis through an RNA-mediated mechanism. For instance, some RNA-binding proteins are sequestered in these foci [29]. However, it is not known which of these proteins shows compromised function that may play a key pathogenic role. On the other hand, abnormal dipeptide repeat proteins (DPRs) arising from repeat-associated non-AUG (RAN) translation—a process discovered in spinocerebellar ataxia type 8 and myotonic dystrophy type 1 [41]—form mostly cytoplasmic inclusions in the brain and spinal cord of patients with *C9ORF72* repeat expansion [3, 30, 42]. Six DPRs can be translated from both sense and antisense expansion transcripts and adjacent intronic sequences of *C9ORF72* [9]. However, the specific roles and mechanisms of each DPR in disease pathogenesis in vivo are largely unknown.

To address these important questions, we generated DNA constructs containing 80 copies of GGXGCX, GGXCGX, or CCXCGX (with X being randomly any one of the four nucleotides) and corresponding transgenic flies that express individual DPRs, such as (GA)_80_, (GR)_80_, and (PR)_80_. When expressed in a cell-type-specific manner, (GR)_80_ and (PR)_80_, but not (GA)_80_, were toxic in neuronal and non-neuronal cells in *Drosophila*. In contrast to a previous report [21], (GR)_80_ was mostly localized throughout the cytosol and did not accumulate in the nucleolus in neurons and wing disc cells. (GR)_80_ suppressed Notch signaling, resulting in cell loss in the wing, a phenotype that was largely suppressed by ectopic expression of Notch. Consistent with these findings, we found lower expression levels of some Notch target genes in iPSC-derived cortical neurons and brain tissues of *C9ORF72* patients. (GR)_80_ toxicity was partially suppressed by co-expression of (GA)_80_, which recruited (GR)_80_ into cytoplasmic inclusions in *Drosophila* cells, HeLa cells, and iPSC-derived human neurons. Thus, the Notch pathway, one of many receptor signaling pathways, is a target of poly(GR) toxicity that in turn can be suppressed by poly(GA) likely through inclusion formation.

## Materials and methods

### Generation and maintenance of transgenic fly lines

Artificial DNA sequences were designed to express 80 repeats of GA, GR, and PR following a Flag tag at the N-terminus. As a control, a stop codon TAA was introduced instead of the ATG codon at the beginning of the open reading frame of GA. The DNA sequences were synthesized (Genewiz) and subcloned into pUASTattB vector between the *Bgl*II and *Xho*I sites. The UAS-(GR)_80_ control construct was generated by site-directed mutagenesis (QuikChange II Site-Directed Mutagenesis Kits, Agilent Technologies), in which the first two nucleotides of the start codon ATG were mutated to TA to form the stop codon TAG (the a5263t_t5264a mutations primer: TCGTTAACAGATCTCCAC CTAGGATTACAAGGACGACGAC). Transgenic flies were made with transgenes *UAS*-*(GA)*_*80*_*control*, *UAS*-*(GA)*_*80*_, *UAS*-*(GR)*_*80*_*control*, *UAS*-*(GR)*_*80*_, and *UAS*-(*PR*)_*80*_ present on the second or third chromosome. The presence of transgenes in the fly was confirmed by sequencing the genomic region amplified by RT-PCR. Primers used were CTGCAACTACTGAAATCTGCCA (forward) and TGTCACACCACAGAAGTAAGGT (reverse). CTGCAACTACTGAAATCTGCCA (forward) was used for sequencing. All flies were raised at 25 °C on a standard diet. *GMR*-*Gal4*, *OK371*-*Gal4*, *UAS*-*GFP/CyO*, *Vg*-*Gal4*, and *w*^*1118*^; *P{NRE*-*EGFP.S}5A* flies were from the Bloomington *Drosophila* Stock Center. *Notch*^*5419*^ mutant and *UAS*-*Notch*^*FL7*^ flies were kindly provided by Dr. S. Artavanis-Tsakonas.

### Quantifying the adult wing notching phenotype

Wings were arbitrarily classified into four groups according to the strength of the phenotype (absent, weak, medium, and strong), as judged from the number of notches and the size of wing area lost.

### Climbing assay and quantification of dendritic branching

*UAS*-*(GA)*_*80*_*control*, *UAS*-*(GA)*_*80*_, *UAS*-*(GR)*_*80*_, and *UAS-(PR)*_*80*_ flies were crossed with *OK371*-*Gal4*, *UAS*-*GFP/CyO* flies at 18 °C to obtain flies expressing dipeptides in motor neurons. For the climbing assay, individual 3-day-old adult flies were placed into a 15-ml polypropylene centrifuge tube (CellTreat Scientific Products). After 1 min, each fly was lightly taped to the bottom of the tube and allowed to climb for 10 s. The climbing distance was scored as the average of five tests for each fly. For quantification of dendritic branching, ddaE sensory neurons were labeled with mCD8-GFP driven by *221*-*Gal4*. The number of dendritic ends was counted at the third-instar larval stage even though the mCD8-GFP signal was reduced by (GR)_80_ and (PR)_80_.

### Immunohistochemistry

Third-instar larval wing imaginal discs, brains or salivary glands were dissected in PBS and fixed in 4 % paraformaldehyde for 20 min at room temperature. After three washes in PBS, samples were permeabilized in PBS containing 0.5 % Triton X-100 (PBT) for 30 min at room temperature and then blocked in 0.5 % goat serum in PBT for 1 h at room temperature. Samples were then incubated with the primary antibody overnight at 4 °C, washed three times with PBT, and incubated with secondary antibody for 2 h at room temperature. The primary antibodies were mouse anti-Flag (Sigma; 1:500) and mouse anti-Wingless (Wg) (Developmental Studies Hybridoma Bank; 1:50). The secondary antibodies were goat anti-mouse Alexa 488 and Alexa 594 (Invitrogen; 1:200). DNA staining was carried out by incubating salivary glands with Quant-iT OliGreen ssDNA reagent (Life Technologies; 1:1000) at room temperature for 5 min. Alternatively, samples were loaded on slides with Vectashield containing 4′,6-diamidino-2-phenylindole (DAPI, Vector laboratories).

### Neuronal cultures and human brain samples

Published iPSC lines from two control subjects and three C9ORF72 carriers [1, 2, 34] were differentiated into cortical neurons as described [2]. Neuronal cultures were aged for 8 weeks before RNA extraction. For (GA)_80_ and (GR)_80_ expression experiments, neuronal cultures from one control line were aged for 4 weeks before transfection. Middle frontal gyrus brain samples from three healthy control subjects and eight *C9ORF72* repeat expansion carriers were obtained from the UCSF Memory and Aging Center. Another three control subjects were from the Mayo Clinic Jacksonville and were used in a recent study [14]. The mean age at death was 78 ± 8 years in six control subjects and 64 ± 7 years in eight *C9ORF72* repeat expansion carriers.

### Quantitative RT-PCR

Total RNA from patient neurons and brain tissues was extracted with the RNeasy kit (Qiagen) according to the manufacturer’s instructions. Total RNA (1000 ng) was reverse transcribed to cDNA with random hexamers and TaqMan reverse transcription reagents (Applied Biosystems). Real-time quantitative PCR was performed with SYBR Green Select Master Mix (Applied Biosystems) on a StepOnePlus system (Applied Biosystems); cyclophilin was used as internal control. Primers used were CGGACATTCTGGAAATGACA (HES1 forward), CATTGATCTGGGTCATGCAG (HES1 reverse), TATCGGAGTTTGGGATTTCG (HEY1 forward), GGGTGATGTCCGAAGACG (HEY1 reverse), TGCCATCGCCAAGGAGTAG (cyclophilin forward), TGCACAGACGGTCACTCAAA (Cyclophilin reverse), cctggatgactcttgggaaa (NFKB1 forward), and tcagccagctgtttcatgtc (NFKB1 reverse). For DPR mRNA analysis, total RNAs were extracted and purified from third-instar larvae tissue with the RNeasy Mini Kit. Ct values for each gene were normalized to *Actin42A* (primers are 5′-TCTTACTGAGCGCGGTTACAG-3′ and 5′-ATGTCGCGCACAATTTCAC-3′). Relative mRNA expression was calculated using the delta–delta Ct method. One pair of primers targeting UAST-attB 3′ UTR (5′-TTCCAACCTATGGAACTGATGA-3′ and 5′-GGTTTTCCTCATTA AAGGCATTC-3′) was selected to detect different dipeptide transcripts.

### Transfection of neurons and HeLa cells and immunofluorescence

For transfection of 4-week-old iPSC-derived neurons and HeLa cells, we used 1.6 µg of plasmid DNA containing HA-(GA)_80_, FLAG-(GR)_80_, or empty vector (pcDNA 3.1, Life Technologies) and Lipofectamine 2000 (Life Technologies). Forty-eight hours after transfection, cells were fixed with 4 % paraformaldehyde and permeabilized with 0.2 % Triton X-100, blocked with 3 % bovine serum albumin, and incubated overnight at 4 °C with primary antibodies against HA (Roche; 1:600) or FLAG (Sigma; 1:1000). After three washes with PBS, the cells were incubated with Alexa Fluor-conjugated secondary antibodies (Invitrogen; 1:300) for 1 h at room temperature and mounted on glass slides with Vectashield HardSet Mounting Medium with DAPI. Immunostained cells were examined by fluorescence microscopy.

### Imaging

The images of adult eyes and wings were acquired with a Nikon DS-Fi1 camera and a Nikon SMZ1500 stereomicroscope. The image of the dorsoventral boundary was acquired with a Nikon D-Eclipse C1. All other images were acquired with a Leica TCS SP5 II laser scanning confocal microscope and Leica LAS AF software. To quantify Wg fluorescence intensity in wing imaginal discs after staining, 30 Z-stack images (step size 0.5 μM) were acquired for each sample. Image-J was used for Z projection, and the integrated intensity of the same region at the dorsoventral boundary of six discs was measured per genotype.

## Results

### Generation of transgenic *Drosophila* models of DPR toxicity

**Fig. 1:**
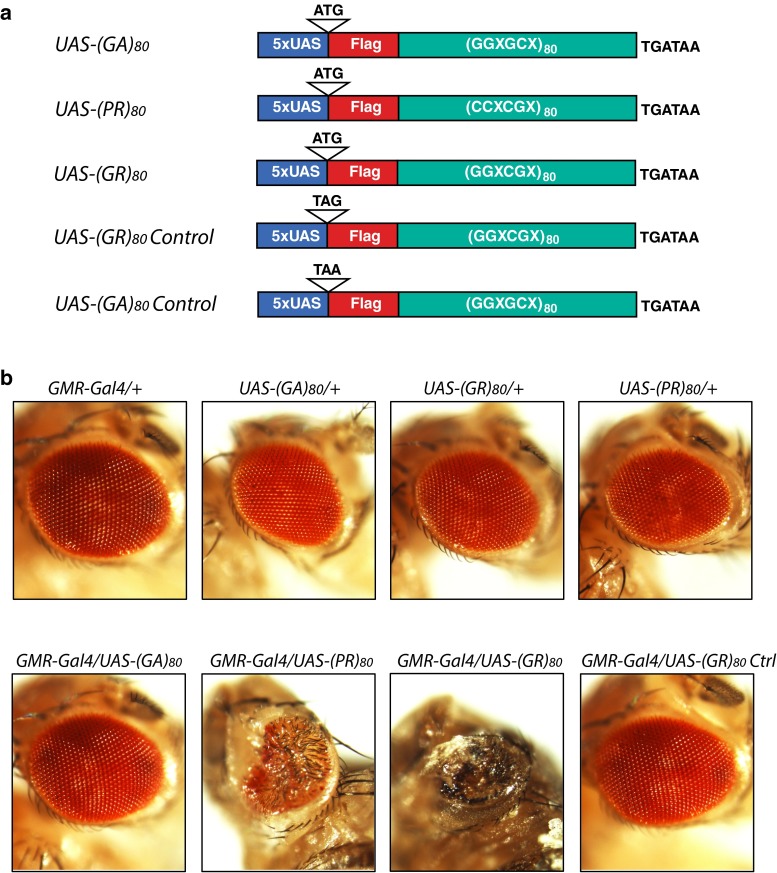
Toxicity of different FTD/ALS-associated dipeptides in the *Drosophila* eye. **a** Schematic representation of DNA constructs that express Flag-tagged (GA)_80_, (PR)_80_, and (GR)_80_ under the control of the UAS elements. In control lines, the AUG start codon was replaced by a UAG or UAA stop codon to block translation. X at the third codon can be randomly any one of the four nucleotides. **b** Representative images of *Drosophila* eyes with different genotypes. Neither *GMR*-*Gal4* nor any of the *UAS* transgenic lines showed an eye phenotype. Expression of (PR)_80_ or (GR)_80_ in the eye by the *GMR*-*Gal4* resulted in grossly deformed eyes

To determine which DPR is toxic in vivo and identify the mechanisms, we generated DNA constructs containing 80 copies of GGXGCX, GGXCGX, or CCXCGX (with X being randomly any one of the four nucleotides) that can be transcribed under the control of UAS elements and translated into (GA)_80_, (GR)_80_, or (PR)_80_, respectively. All constructs contained the CCACC consensus Kozak sequence adjacent to the ATG start codon and a DNA sequence encoding the Flag tag at the N-terminus of DPRs (Fig. 1a; Tab. S1). To generate transgenic fly lines, we used the PhiC31 integrase-mediated site-specific integration system [5] to ensure equal transcription of different DPR constructs in a cell-type specific manner when a unique Gal4 driver is used [8].

To dissect the toxicity of DPRs, we first used *GMR*-*Gal4*, which drives ectopic gene expression mostly in the eye, a widely used model for studying neurodegeneration [6, 24]. Expression of (PR)_80_ and (GR)_80_, but not (GA)_80_, driven by *GMR*-*Gal4* resulted in a mostly adult lethal phenotype and caused a drastic deformation of eyes in all flies at the pupal stage; neither the *GMR*-*Gal4* nor any of the UAS transgenic lines alone had any eye phenotype (Fig. 1b). More than 200 flies were examined for each genotype. To confirm that different DPRs were expressed in the eye tissue, we performed RT-PCR analysis using primers specific to the 3′UTR sequence common to all DPR constructs. Indeed, all DPR mRNAs were expressed, although at different levels (Fig. S1), which is probably due to differences in mRNA stability. The stability, degradation, and biophysical properties of (GA)_80_ proteins may also differ from those of (PR)_80_ and (GR)_80_. Indeed, immunostaining analysis with Flag tag-specific antibody showed all three DPRs were expressed when *OK371*-*Gal4* was used (Fig. S2a–c), which drives target gene expression in cholinergic neurons including motor neurons. However, unlike (GR)_80_ (Fig. S2b) and (PR)_80_ (Fig. S2c), (GA)_80_ forms distinct inclusions, mostly one in each neuron (Fig. S2a). Thus, although the extent of toxicity between different DPRs cannot be compared directly, lack of (GA)_80_ toxicity is correlated with its inclusion formation, while more diffuse (GR)_80_ and (PR)_80_ cause drastic phenotypes under the same experimental condition.

**Fig. 2:**
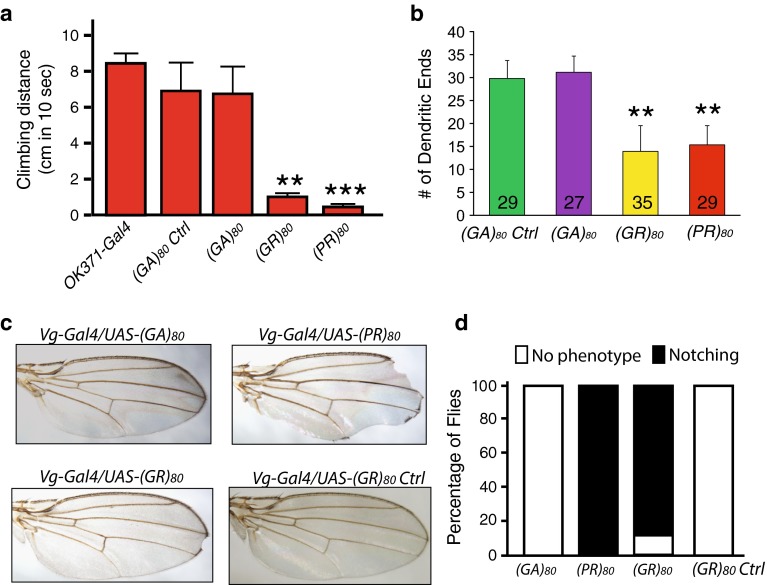
(GR)_80_ is toxic in neuronal and non-neuronal cells in *Drosophila*. **a** Effects of (GA)_80_, (GR)_80_, and (PR)_80_ on locomotor activity of adult flies. (GA)_80_, (GR)_80_, and (PR)_80_ were expressed in motor neurons by *OK371*-*Gal4* at 18 °C, and surviving 3-day-old adult flies (10 flies of each genotype) were tested for climbing activity. Forty *OK371*-*Gal4* flies and 10 flies expressing *(GA)*_*80*_ mRNA but with a stop codon instead of the ATG start codon (see Table S1) were also examined. Values are mean ± SEM. **p value <0.01, ***p value <0.001, by Student’s *t* test. **b** Effects of (GA)_80_, (GR)_80_, and (PR)_80_ expression on dendritic branching of ddaE sensory neurons. ddaE neurons were labeled with mCD8-GFP driven by 221-Gal4. The number of neuron analyzed for each genotype is listed on each column. Values are mean ± SD. **p value <0.01, by single-factor ANOVA. **c** Effects of (GA)_80_, (GR)_80_, and (PR)_80_ on the survival of wing margin cells. The expression of (GR)_80_, and (PR)_80_ resulted in the wing notching phenotype. **d** The percentage of flies with or without wing notching phenotype is shown for each genotype. More than 300 flies of both sexes were scored for each genotype

To further exclude the possibility that (GR)_80_ toxicity might be caused by transcription of GC-rich RNAs in vivo, we also generated transgenic fly lines that expressed the same *(GR)*_*80*_ mRNA except that the AUG start codon was replaced with the UAG stop codon (Fig. 1a). Expression of this control construct from the same genomic locus under the same Gal4 driver was not toxic (Fig. 1b). Thus, (GR)_80_ and (PR)_80_ proteins are highly toxic when overexpressed in the *Drosophila* eye by the *GMR*-*Gal4*.

### (GR)_80_ is toxic in neuronal and non-neuronal cells in *Drosophila*

Since *GMR*-*Gal4* drives target gene expression at a high level, the drastic toxicity of (GR)_80_ and (PR)_80_ in the eye should be considered in the context of their overexpression. Therefore, we searched for Gal4 drivers that would give rise to a more moderate phenotype, enabling us to study the mechanism. Expression of the (GA)_80_ construct by various Gal4s did not result in any obvious phenotypes (Table S2). However, expression of (GR)_80_ and (PR)_80_ in all cells by *tubulin*-*Gal4*, in all neurons by *elav*-*Gal4*, or in motor neurons by *D42* or *OK371*-*Gal4* at 25 °C resulted in a mostly lethal phenotype (Table S2). The strength of Gal4 drivers is dependent on temperature. Lower expression of (GR)_80_ and (PR)_80_ by *OK371*-*Gal4* at 18 °C caused a semi-lethal phenotype (Table S2), and surviving adult flies had greatly reduced locomotor activity (Fig. 2a). Moreover, expression of (GR)_80_ and (PR)_80_ in ddaE sensory neurons decreased dendritic branching (Fig. 2b). (GR)_80_-control was not used in these experiments because it did not produce any eye phenotype (Fig. 1b). Expression of (GR)_80_, but not the (GR)_80_ control construct, by *Vg*-*Gal4*, which drives gene expression in the dorsoventral boundary and some other cells of the wing imaginal discs [11], gave rise to wing margin defects in 90 % of flies of both sexes at 25 °C (Fig. 2c, d). Thus, (GR)_80_ is toxic in multiple neuronal and non-neuronal cell types in vivo.

### (GR)_80_ genetically interacts with *Notch*

**Fig. 3:**
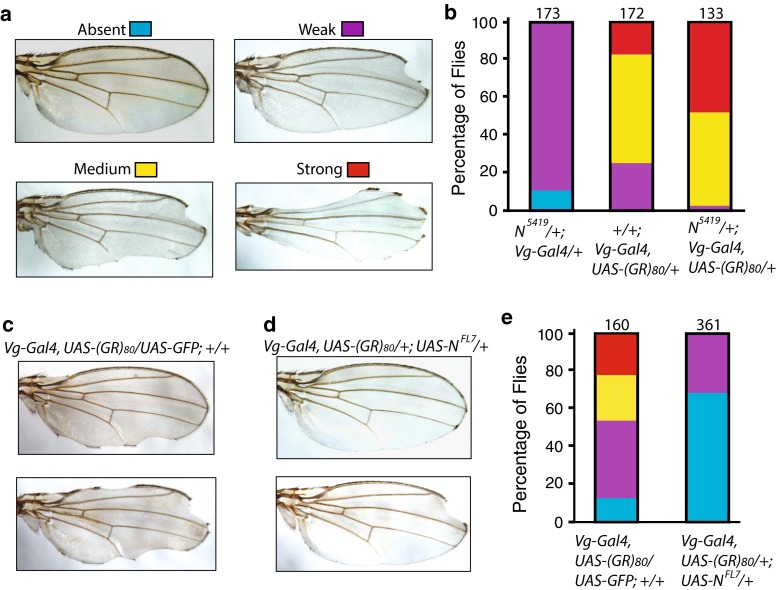
Notch expression suppresses (GR)_80_ toxicity. **a** Representative images of *Drosophila* wings with margin defects of different severities. The image of a normal wing was from a *UAS*-*(GR)*_*80*_/+ fly; other wing images were from *N* ^*5419*^/+; *Vg*-*Gal4, UAS*-*(GR)*_*80*_/+ flies. **b** Quantification of wing margin defects in female flies heterozygous for the *N* ^*5419*^ allele, expressing (*GR)*_*80*_ driven by *Vg*-*Gal4* recombined to the same second chromosome and expressing (*GR)*_*80*_ on the *N* ^*5419*^/+ background by *Vg*-*Gal4*. Only female flies were examined, and the total number of flies for each genotype from three experiments is listed above each column. **c** Representative images of wing margin defects in *Vg*-*Gal4, UAS*-*(GR)*_*80*_ */*+ flies. **d** Representative wing images of *Vg*-*Gal4*, *UAS*-*(GR)*_*80*_ */*+; *UAS*-*N* ^*FL7*^ flies showing suppression of the wing margin defects by ectopic expression of full-length Notch. *UAS*-*GFP* was used as the control for *UAS*-*N* ^*FL7*^. **e** Quantification of Notch suppression of wing margin defects caused by (GR)_80_. The total number of flies of both sexes examined for each genotype from 3 experiments is listed above each column

Misregulation of several signaling pathways results in distinct wing defects [4]. However, the mild notching defects at or near the tip of the wing caused by (GR)_80_ are remarkably similar to the defect due to partial loss of Notch activity, as in flies heterozygous for the *N*^*5419*^ allele [20]. Therefore, we investigated the genetic interaction between *Notch* and (GR)_80_. We grouped wing notching phenotypes by their severity: absent, weak, medium, and strong (Fig. 3a). Since the *Notch* gene is located on the X chromosome, we examined only female flies in this genetic interaction experiment. About 90 % of female flies heterozygous for the *N*^*5419*^ allele had a weak wing margin defect (Fig. 3b). To facilitate the genetic interaction study, *Vg*-*Gal4* and *UAS*-*(GR)*_*80*_ were recombined onto the same second chromosome. The wing notching phenotype in the resulting fly line (Fig. 3b) was stronger than that in flies transheterozygous for *Vg*-*Gal4* and *UAS*-*(GR)*_*80*_ (Fig. 2c). When this line was examined in the *N*^*5419*^*/*+ background, the wing notching phenotype was substantially stronger than would be expected from an additive effect of (GR)_80_ and *N*^*5419*^*/*+: 18 % of (GR)_80_ flies versus 47 % of (GR)_80_ flies in the *N*^*5419*^*/*+ background showed a strong wing margin phenotype (*p* < 0.05, *n* = 3), raising the possibility that the two genes genetically interact (Fig. 3b). These results suggest that (GR)_80_ compromises the Notch signaling pathway. Indeed, (GR)_80_ toxicity was largely suppressed by ectopic expression of full-length Notch; a wing notching phenotype was absent in 67 % of *(GR)*_*80*_ flies when Notch was co-expressed but in only 12 % of *(GR)*_*80*_ flies when GFP was co-expressed (*p* < 0.001, *n* = 3) (Fig. 3c–e). (PR)_80_ toxicity was not suppressed by Notch expression, and PR_80_ expression in *N*^*5419*^*/*+ background did not significantly enhance the PR_80_ wing defect (not shown), suggesting divergent pathogenic mechanisms of these two DPRs. To ensure that the suppression by Notch was not due to the dilution of Gal4 caused by a second copy of the UAS transgene, flies expressing both (GR)_80_ and GFP by *Vg*-*Gal4* were used as controls in this experiment (Fig. 3c). These genetic analyses suggest that the Notch pathway is a major target of (GR)_80_ toxicity in vivo.

### (GR)_80_ suppresses Notch signaling

**Fig. 4:**
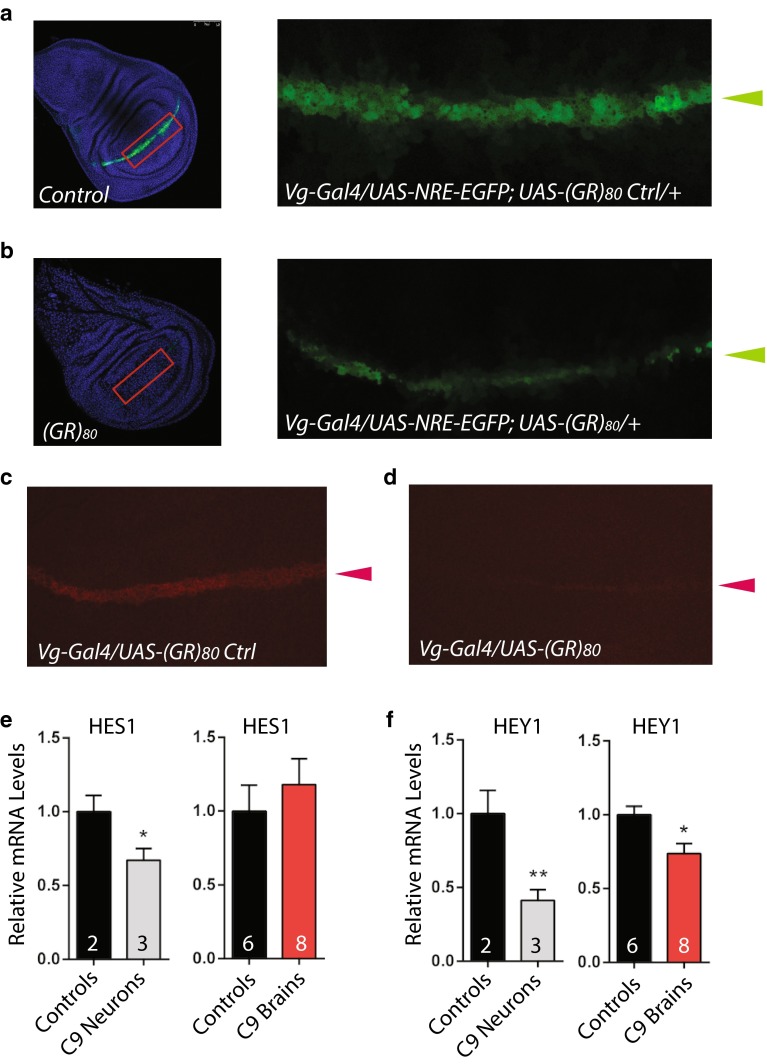
(GR)_80_ downregulates notch signaling in *Drosophila*. GFP expression controlled by the Notch-responsive element (*NRE*) at the dorsoventral boundary (*green arrowheads*) of a control wing disc (**a**) and a wing disc expressing (GR)_80_ driven by *Vg*-*Gal4* (**b**). The areas indicated by *red rectangles* are enlarged and presented as the *two right panels*. Wg immunostaining at the dorsoventral boundary (*red arrowheads*) of a control wing disc (**c**) and a wing disc expressing (GR)_80_ (**d**). Expression levels of the Notch targets *HES1* (**e**) and *HEY1* (**f**) in iPSC-derived neurons and brain tissues of subjects with C9ORF72 repeat expansion. The number of iPSC lines or brain samples analyzed is indicated in each column. Values are mean ± SEM. *p value <0.05, **p value <0.01 by Student’s *t* test

To confirm (GR)_80_ indeed suppresses Notch signaling, we expressed a Notch activity reporter, *UAS*-*Notch Response Element (NRE)*-*EGFP* [33], in the wing disc cells by *Vg*-*Gal4*. Notch signaling was generally lower in all cells at the dorsoventral boundary that express *UAS*-*(GR)*_*80*_ (Fig. 4b) than in control flies expressing *UAS*-*(GR)*_*80*_*Control* (Fig. 4a). The dorsoventral boundary remained intact, and no obvious cell loss was observed, as judged from caspase-3 immunostaining. To further confirm this finding, we examined the expression level of endogenous Wingless (Wg) at the dorsoventral boundary of the wing disc, which is a direct target of Notch signaling [12]. Indeed, (GR)_80_ significantly suppressed endogenous Wg expression (Fig. 4c, d). Although we cannot rule out the possibility that (GR)_80_ affects the Wg level through other mechanisms, this result is consistent with the finding that (GR)_80_ decreases endogenous Notch signaling.

Poly(GR) proteins have been detected mostly in the cytoplasmic inclusions in brain tissues of patients with C9ORF72 repeat expansion [3, 30, 42], thus it is possible that Notch signaling in patient neurons may also be compromised. In recent years, patient-specific iPSCs have been used as a powerful model for many neurodegenerative diseases [18, 38, 39]. We differentiated iPSC lines from two control and three subjects with C9ORF72 repeat expansions [1, 2, 34] into 8-week-old postmitotic neurons of cortical lineage and found reduced expression of the Notch target genes *HES1* (Fig. 4e) and *HEY1* (Fig. 4f). *HEY1*, one of the target genes more sensitive to Notch level [35], showed reduced expression in brain tissues of *C9ORF72* patients (Fig. 4f). The expression of Notch target NFKB1 was also lower in both iPSC-derived neurons and brain tissues of C9ORF72 patients (not shown). Although these results are correlative, Notch signaling seems to be compromised in patient cells as well.

### (GA)_80_ partially suppresses (GR)_80_ toxicity through inclusion formation

To further examine the effects of (GR)_80_ on Notch signaling, we examined the subcellular localization of (GR)_80_ expressed in a subset of wing disc cells by *Vg*-*Gal4* (Fig. S2d). Immunostaining with either anti-Flag antibody or anti-GR antibody revealed that this DPR was localized in the cytoplasm (Fig. S2e and g), suggesting that (GR)_80_ interferes with the Notch signaling pathway in the cytoplasm. In both wing disc cells (Fig. S2f) and salivary gland cells (Fig. S3d), (PR)_80_ also seemed to be mostly cytoplasmic.

**Fig. 5:**
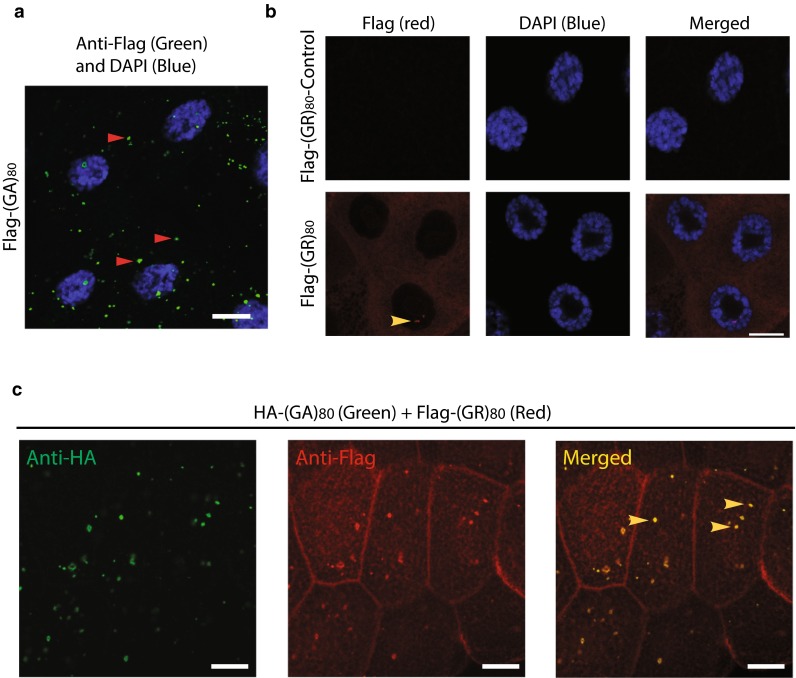
Subcellular localization of (GR)_80_ and (GA)_80_. **a** Flag-tagged (GA)_80_ forms inclusions mostly in the cytosol of *Drosophila* salivary gland cells. Some inclusions are highlighted by *red arrowheads*. **b** (GR)_80_ is largely present throughout the cytoplasm of salivary gland cells, and their nucleoli are larger than those of cells expressing the (GR)_80_-control construct. These are confocal images, and one or two (GR)_80_-positive dots (*yellow arrowhead*) were observed on chromatin in each cell at different confocal planes. *Scale bar* 20 μm. **c** HA-tagged (GA)_80_ recruits Flag-tagged (GR)_80_ into cytoplasmic inclusions when the two are co-expressed. All inclusions contain both (GA)_80_ and (GR)_80_ (some are indicated by *yellow arrowheads*)

Since both poly(GA) and poly(GR) proteins are concomitantly expressed in patient cells [3, 30, 42], we first co-expressed (GA)_80_ and (GR)_80_ in *Drosophila* salivary gland cells, which are large and facilitate imaging analysis. (GA)_80_ by itself formed mostly cytoplasmic inclusions (Fig. 5a), while (GR)_80_ was localized throughout the cytoplasm in salivary gland cells (Fig. 5b), as in wing disc cells (Fig. S2e). This subcellular distribution of (GR)_80_ in salivary gland cells was confirmed with the anti-GR antibody (Fig. S3e). We also noticed one or two small (GR)_80_-positive dots on chromatin in each salivary gland cell (Fig. 5b, Fig. S3e), as well as in *Drosophila* motor neurons (Fig. S3f). Their nature and significance remain to be determined. Shorter GR forms have been reported to accumulate in nucleoli of cultured cells [21], but we found no significant presence of (GR)_80_ in nucleoli of salivary gland cells, even though their nucleoli were enlarged (Fig. 5b). But unexpectedly, in the presence of HA-(GA)_80_ (Fig. 5c, left panel), a portion of Flag-(GR)_80_ formed cytoplasmic inclusions as well (Fig. 5c, middle panel), in contrast to the diffuse cytoplasmic localization when only Flag-(GR)_80_ was expressed (Fig. 5b). Because (GA)_80_ alone forms cytoplasmic inclusions (Fig. 5a), co-localization of Flag-(GR)_80_ and HA-(GA)_80_ suggested that (GA)_80_ recruits (GR)_80_ into these inclusions (Fig. 5c, right panel).

**Fig. 6:**
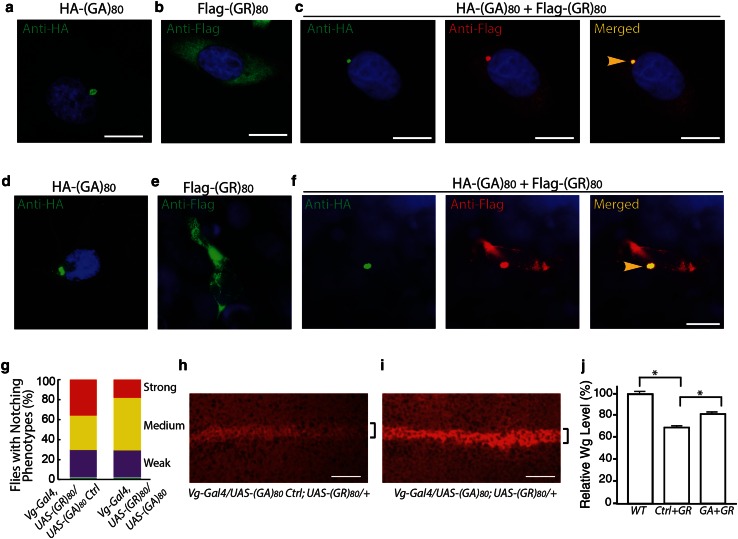
(GA)_80_ suppresses (GR)_80_ toxicity through inclusion formation. **a** HA-tagged (GA)_80_ forms inclusion in HeLa cells. **b** (GR)_80_ expression alone in HeLa cells shows diffuse cytoplasmic localization. **c** When (GA)_80_ and (GR)_80_ are co-expressed in HeLa cells, (GR)_80_ is recruited into (GA)_80_ inclusions (*yellow arrowhead*). **d** HA-tagged (GA)_80_ forms inclusion in iPSC-derived human neurons. **e** (GR)_80_ expression alone in iPSC-derived human neurons shows cytoplasmic localization. **f** When (GA)_80_ and (GR)_80_ are co-expressed in iPSC-derived human neurons, (GR)_80_ is recruited into (GA)_80_ inclusions (*yellow arrowhead*). *Scale bar* 10 μm. **g** (GA)_80_ partially suppresses (GR)_80_ toxicity in wing disc cells, resulting in a less severe wing notching phenotype. In this experiment, *Vg*-*Gal4* and *UAS*-*(GR)*_*80*_ were recombined onto the same chromosome. Wg expression at the dorsoventral boundary of wing discs expressing *UAS*-*(GA)*_*80*_ *control* (Table S1) and *UAS*-*(GR)*_*80*_ (**h**) or both *UAS*-*(GA)*_*80*_ and *UAS*-*(GR)*_*80*_ (**i**). *UAS*-*(GR)*_*80*_ on the third chromosome. The brackets indicate the dorsoventral boundary. **j** Wg expression levels in wing discs of flies with genotypes described in panels **h** and **i**. *Scale bar* in *panels* **a**–**f**, **h**, **i**: 20 μm. Values are mean ± SEM. *p value <0.05 by single-factor ANOVA

Similarly, in human HeLa cells, (GA)_80_ alone formed inclusions (Fig. 6a), while (GR)_80_ alone was localized throughout the cytoplasm without any detectable accumulation in the nucleus (Fig. 6b). (GA)_80_ inclusions seemed to be aggresomes surrounded by vimentin and were often close to the nucleus (Fig. S4a) and positive for p62 (Fig. S4b). When (GA)_80_ and (GR)_80_ were co-expressed, some (GR)_80_ co-localized with (GA)_80_ inclusions in all HeLa cells we examined (Fig. 6c). Moreover, in iPSC-derived human neurons, (GA)_80_ recruited (GR)_80_ into cytoplasmic inclusions as well (Fig. 6d–f). These findings raise the possibility that (GA)_80_ protein is protective and can sequester highly toxic poly(GR) protein into inclusions. Indeed, (GA)_80_ expression partially suppressed the (GR)_80_-induced cell-loss phenotype at the wing margin (Fig. 6g) and correspondingly increased the Notch signaling, as indicated by the elevated expression of Wg at the dorsoventral boundary of the wing disc (Fig. 6h–j). (GA)_80_ expression did not suppress the (GR)_80_-induced eye phenotype (not shown), presumably because *GMR*-*Gal4* is a very strong driver so that the level of non-aggregated (GR)_80_ remained high. Thus, (GA)_80_ partially suppresses (GR)_80_ toxicity in vivo by sequestering (GR)_80_ into inclusions.

## Discussion

This study shows that poly(GR) proteins are toxic in various neuronal and non-neuronal cell types in vivo under defined experimental conditions, consistent with several reports published during the preparation of our manuscript [21, 28, 36, 37]. However, in contrast to some of these studies, in which smaller poly(GR) proteins provided to cultured cells migrate to and significantly accumulate in the nucleolus, we found that (GR)_80_ is mostly localized throughout the cytoplasm in vivo and does not accumulate in the nucleolus. Thus, it is possible that DPRs of different lengths have diverse biological properties and pathogenic mechanisms.

We found that (GR)_80_ compromises Notch signaling and that ectopic expression of Notch partially suppresses (GR)_80_ toxicity in *Drosophila*. The expression levels of DPRs in many experimental systems, including ours in this study, are likely much higher than in human neurons, especially before disease onset. Thus, detrimental effects of low levels of DPRs in postmitotic neurons over a long period remain to be examined, and some of the mechanisms may differ from those found in overexpression studies. Nonetheless, Notch signaling also seems to be downregulated in iPSC-derived neurons and postmortem brain tissues of subjects with C9ORF72 repeat expansion; however, this result is correlative and should be interpreted cautiously. Together, our results raise the possibility that some key receptor signaling pathways are compromised in C9ORF72 FTD/ALS patients. The Notch signaling pathway is subject to complex regulation at multiple steps [17]. It would be interesting to determine exactly how (GR)_80_ and possibly other poly(GR) proteins of different lengths affect the Notch pathway or other receptor signaling pathways. The relative contributions of the Notch pathway and other affected signaling pathways to dysfunction of patient neurons also remain to be examined.

Another pathological feature of (GR)_80_ was the presence of small (GR)_80_-positive dots on chromatin in postmitotic cells in *Drosophila* (Fig. 5b, Fig. S3e, f) but not in dividing HeLa cells (Fig. 6b). The nature and significance of these (GR)_80_-positive structures on chromatin are unknown. If similar structures can be found in diseased neurons of C9ORF72 FTD/ALS patients, our fly model would be a useful tool for further investigation. It is also worth noting that *Drosophila* cells expressing (GR)_80_ have enlarged nucleoli even in the absence of detectable accumulation of (GR)_80_ in this subcellular compartment. Various cellular stresses induce specific changes in nucleolar morphology and composition in many cell types, including postmitotic neurons [7, 19]. Thus, nucleolar defects in C9ORF72 FTD/ALS can be caused by indirect mechanisms other than direct actions of G_4_C_2_ repeat RNAs or DPRs in the nucleolus.

Subjects with C9ORF72 repeat expansion often live for decades without symptoms, and DPR inclusions are mostly cytoplasmic and more abundant in brain regions without neurodegeneration [3, 30, 42]. Our finding that (GA)_80_ suppresses (GR)_80_ toxicity by sequestering (GR)_80_ into cytoplasmic inclusions suggests that different DPRs have diverse roles in neuronal cell death. Although transient overexpression of poly(GA) alone in cultured cells can be detrimental [26, 40], our findings suggest that aggregation-prone poly(GA) proteins may also have an in vivo neuroprotective function in early stages of disease by sequestering highly toxic poly(GR) into inclusions. We speculate that in the early stage of disease, poly(GR) is efficiently sequestered by poly(GA) inclusions and degraded, likely through the autophagy pathway. But with disease progression, overall DPR toxicity in some neurons may increase due to elevated levels of non-aggregated poly(GR). Thus, sequestration of poly(GR) proteins and other approaches to alleviate their detrimental effects on receptor signaling pathways are potential therapeutic avenues.

## Electronic supplementary material

Supplementary material 1 (DOCX 684 kb)

Supplementary material 2 (PDF 65 kb)

Supplementary material 3 (PDF 166 kb)

Supplementary material 4 (PDF 125 kb)

Supplementary material 5 (PDF 107 kb)
