# Supplementary material for: FTD/ALS-associated poly(GR) protein impairs the Notch pathway and is recruited by poly(GA) into cytoplasmic inclusions
Source: Acta Neuropathol. 2015 Jun 2;130(4):525–35. doi: 10.1007/s00401-015-1448-6 (PMC4575383; doi:10.1007/s00401-015-1448-6)

**Supplemental materials**

**Figure Legends**

**Fig. S1. Different DPRs are expressed in *Drosophila* larvae.** To demonstrate different DPR mRNAs are expressed driven by *GMR-Gal4*, primers specific to the common 3’UTR sequence in the UAS vector were used for RT-PCR analysis of DPR mRNA levels. Because eye defects caused by (GR)_80_ or (PR)_80_ are so drastic, *Drosophila* larvae were used for the RT-PCR analysis.

**Fig. S2**. Assessing the expression of different DPRs in motor neurons and wing disc cells. **a** Flag-(GA)_80_. Scale bar: 5 μm. **b** Flag-(GR)_80_. Scale bar: 5 μm. **c** Flag-(PR)_80_. Scale bar: 5 μm. In panels a-c, the expression of DPRs in motor neurons is driven by *OK-371-Gal4*. Green: anti-Flag, blue: DAPI. **d** (GR)_80_ is expressed in a subset of cells in the wing disc by *Vg-Gal4*. Scale bar: 50 μm. **e** (GR)_80_ is localized mostly in the cytosol of wing disc cells, as revealed by immunostaining with anti-Flag antibody. Scale bar: 5 μm. **f** (PR)_80_ is mostly localized in the cytosol of wing disc epithelial cells. Scale bar: 5 μm. **g** Cytoplasmic localization of (GR)_80_ as shown by anti-GR antibody. **h** Immunostaining with anti-GR antibody of a wing disc without (GR)_80_ expression, demonstrating the specificity of the antibody. Blue in panels a-c, e and f: DAPI.

**Fig. S3**. Expression of different DPRs in salivary gland cells. **a** *Fkh-Gal4* drives GFP expression in salivary gland cells. Free GFP accumulates in the nucleus. Scale bar: 50 μm. **b** Negative control for the anti-Flag antibody showing the absence of immunostaining signal in salivary gland cells without any Flag tagged DPR expression. Scale bar: 50 μm. **c** GR localization shown by immunostaining with anti-Flag antibody in salivary gland cells expressing (GR)_80_. Scale bar: 50 μm. **d** (PR)_80_ seems to be mostly localized in the cytosol as well. (PR)_80_ toxicity greatly reduces the size of salivary gland. Scale bar: 50 μm. **e** Immunostaining with anti-GR antibody shows the mostly cytoplasmic localization of (GR)_80_ in salivary gland cells. Blue: DAPI. The yellow arrowhead indicates a (GR)_80_-positive dot on chromatin. Scale bar: 20 μm. **f** The yellow arrowhead indicates (GR)_80_-positive dots on chromatin in larval motor neurons. Scale bar: 5 μm.

**Fig. S4**. Characterization of (GA)_80_ inclusions. **a** Two examples of HeLa cells with HA-(GA)_80_ inclusions that are surrounded by vimentin. Scale bar: 20 μm. **b** (GA)_80_ inclusion in HeLa cells is also p62-positive, as indicated by the yellow arrowhead. Scale bar: 20 μm.

**Tab. S1**. Nucleotide sequences encoding different DPRs with the Flag tag.

| Flag-(GA)_80_ | CCACCATGGATTACAAGGACGACGACGATAAGTTAGGGGCTGGAGCGGGAGCGGGTGCTGGGGCTGGGGCAGGAGCAGGCGCAGGGGCAGGCGCTGGAGCGGGAGCCGGTGCTGGAGCCGGAGCTGGAGCGGGCGCAGGTGCGGGTGCTGGTGCCGGGGCTGGCGCTGGCGCTGGAGCTGGTGCTGGGGCAGGGGCCGGTGCTGGAGCAGGCGCTGGAGCTGGCGCAGGAGCAGGGGCTGGTGCTGGAGCTGGAGCAGGCGCTGGAGCCGGTGCTGGTGCTGGAGCAGGTGCTGGAGCTGGAGCAGGCGCTGGAGCTGGCGCAGGCGCAGGAGCCGGAGCAGGTGCTGGCGCTGGAGCTGGTGCAGGCGCAGGAGCAGGTGCTGGAGCCGGAGCAGGTGCTGGAGCTGGTGCGGGAGCTGGAGCAGGTGCCGGAGCTGGTGCGGGAGCTGGTGCGGGAGCAGGCGCAGGTGCTGGAGCCGGAGCTGGCGCAGGTGCGGGCGCAGGTGCTGGAGCTTGATAA |
| --- | --- |
| Flag-(GR)_80_ | CCACCATGGATTACAAGGACGACGACGATAAGTTAGGTCGTGGACGTGGACGAGGTCGGGGTCGCGGACGAGGACGCGGTCGTGGACGAGGTCGTGGACGTGGTCGAGGACGTGGTCGTGGTCGTGGACGAGGTCGTGGACGTGGACGAGGTCGGGGACGTGGTCGTGGTCGTGGACGAGGTCGCGGACGAGGACGCGGTCGTGGACGAGGTCGTGGACGTGGTCGAGGACGAGGTCGTGGTCGTGGACGTGGACGAGGTCGTGGACGCGGTCGTGGTCGTGGACGAGGTCGTGGACGTGGACGAGGTCGTGGACGTGGTCGAGGTCGAGGACGCGGACGAGGTCGTGGTCGTGGACGTGGACGAGGTCGAGGACGAGGTCGTGGACGCGGACGAGGTCGTGGACGTGGTCGGGGACGTGGACGAGGTCGCGGACGTGGTCGGGGACGTGGTCGGGGACGAGGTCGAGGTCGTGGACGCGGACGTGGTCGAGGTCGGGGTCGAGGTCGTGGACGTTGATAA |
| Flag-(PR)_80_ | CCACCATGGATTACAAGGACGACGACGATAAGTTACCCAGACCTCGTCCACGACCTCGTCCCAGACCACGTCCACGTCCTCGCCCACGTCCTCGTCCACGTCCCAGGCCACGACCTCGACCAAGACCTAGGCCACGTCCTAGACCTCGACCGCGTCCTCGACCTAGACCGCGTCCTAGGCCAAGACCTAGACCGAGGCCACGACCTAGACCGAGACCACGACCAAGACCACGACCTCGTCCAAGGCCTCGTCCCCGTCCTAGACCTCGTCCCCGTCCACGTCCGAGACCACGCCCACGTCCTCGTCCGAGACCTCGGCCACGACCTAGACCTAGGCCACGACCTCGCCCAAGACCTAGGCCACGTCCTCGTCCGAGACCTAGGCCACGTCCTCGTCCAAGACCTCGCCCAAGACCTAGGCCACGCCCTAGACCAAGGCCTAGACCAAGGCCAAGACCTCGTCCACGTCCACGTCCTCGTCCGAGGCCAAGACCTCGACCTCGTCCTCGTCCACGTTGATAA |
| Control for (GR)_80_ | CCACCTAGGATTACAAGGACGACGACGATAAGTTAGGGGCTGGAGCGGGAGCGGGTGCTGGGGCTGGGGCAGGAGCAGGCGCAGGGGCAGGCGCTGGAGCGGGAGCCGGTGCTGGAGCCGGAGCTGGAGCGGGCGCAGGTGCGGGTGCTGGTGCCGGGGCTGGCGCTGGCGCTGGAGCTGGTGCTGGGGCAGGGGCCGGTGCTGGAGCAGGCGCTGGAGCTGGCGCAGGAGCAGGGGCTGGTGCTGGAGCTGGAGCAGGCGCTGGAGCCGGTGCTGGTGCTGGAGCAGGTGCTGGAGCTGGAGCAGGCGCTGGAGCTGGCGCAGGCGCAGGAGCCGGAGCAGGTGCTGGCGCTGGAGCTGGTGCAGGCGCAGGAGCAGGTGCTGGAGCCGGAGCAGGTGCTGGAGCTGGTGCGGGAGCTGGAGCAGGTGCCGGAGCTGGTGCGGGAGCTGGTGCGGGAGCAGGCGCAGGTGCTGGAGCCGGAGCTGGCGCAGGTGCGGGCGCAGGTGCTGGAGCTTGATAA |
| Control for (GA)_80_ | CCACCTAAGATTACAAGGACGACGACGATAAGTTAGGGGCTGGAGCGGGAGCGGGTGCTGGGGCTGGGGCAGGAGCAGGCGCAGGGGCAGGCGCTGGAGCGGGAGCCGGTGCTGGAGCCGGAGCTGGAGCGGGCGCAGGTGCGGGTGCTGGTGCCGGGGCTGGCGCTGGCGCTGGAGCTGGTGCTGGGGCAGGGGCCGGTGCTGGAGCAGGCGCTGGAGCTGGCGCAGGAGCAGGGGCTGGTGCTGGAGCTGGAGCAGGCGCTGGAGCCGGTGCTGGTGCTGGAGCAGGTGCTGGAGCTGGAGCAGGCGCTGGAGCTGGCGCAGGCGCAGGAGCCGGAGCAGGTGCTGGCGCTGGAGCTGGTGCAGGCGCAGGAGCAGGTGCTGGAGCCGGAGCAGGTGCTGGAGCTGGTGCGGGAGCTGGAGCAGGTGCCGGAGCTGGTGCGGGAGCTGGTGCGGGAGCAGGCGCAGGTGCTGGAGCCGGAGCTGGCGCAGGTGCGGGCGCAGGTGCTGGAGCTTGATAA |

Note: The consensus Kozak sequence CCACC sequence is highlighted in blue. The ATG start codon and stop codons are highlighted in red. The DNA sequence encoding the Flag tag is highlighted in green.

**Tab. S2**. Effects of (GA)_80_, (GR)_80_ and (PR)_80_ expression driven by different Gal4s.


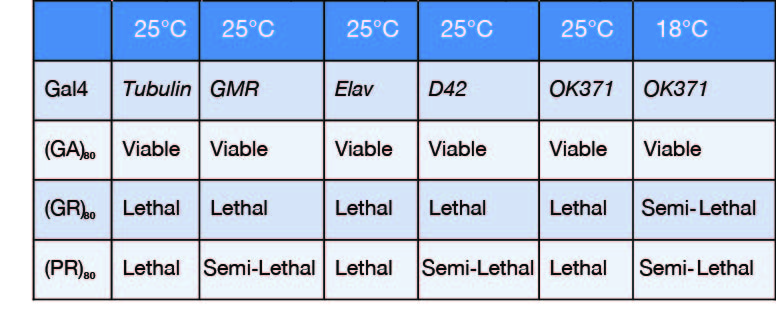

Supplement: Supplementary file 1 — Supplementary material 1 (DOCX 684 kb) [file 401_2015_1448_MOESM1_ESM.docx]
