# Supplementary figures and images for: FTD/ALS-associated poly(GR) protein impairs the Notch pathway and is recruited by poly(GA) into cytoplasmic inclusions
Source: Acta Neuropathol. 2015 Jun 2;130(4):525–35. doi: 10.1007/s00401-015-1448-6 (PMC4575383; doi:10.1007/s00401-015-1448-6)

Yang et al. Figure S1

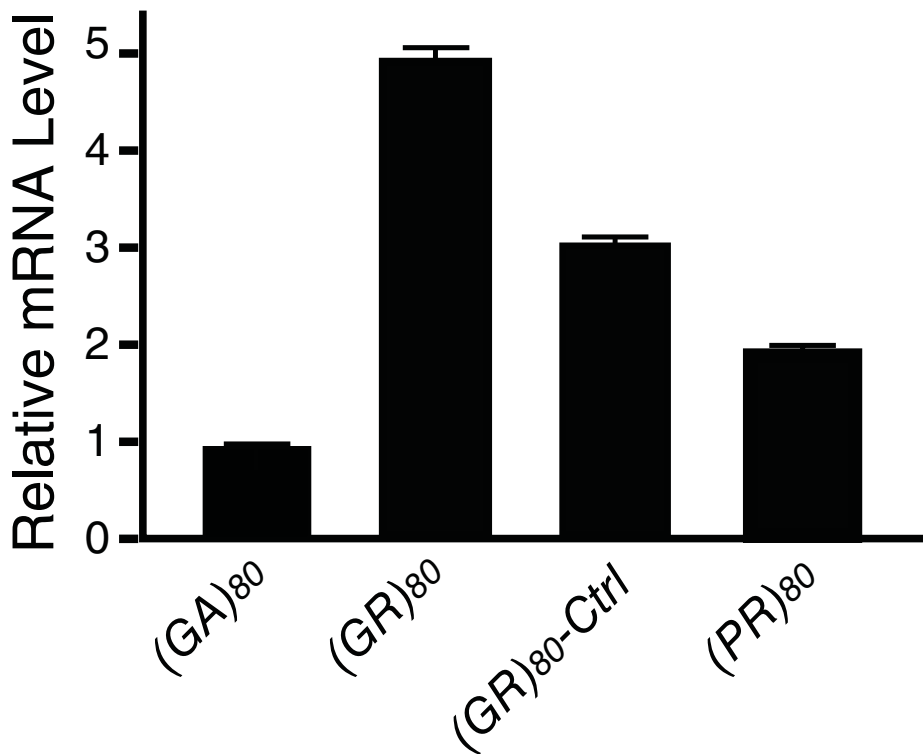

Supplement: Supplementary file 2 — Supplementary material 2 (PDF 65 kb) [file 401_2015_1448_MOESM2_ESM.pdf]

a

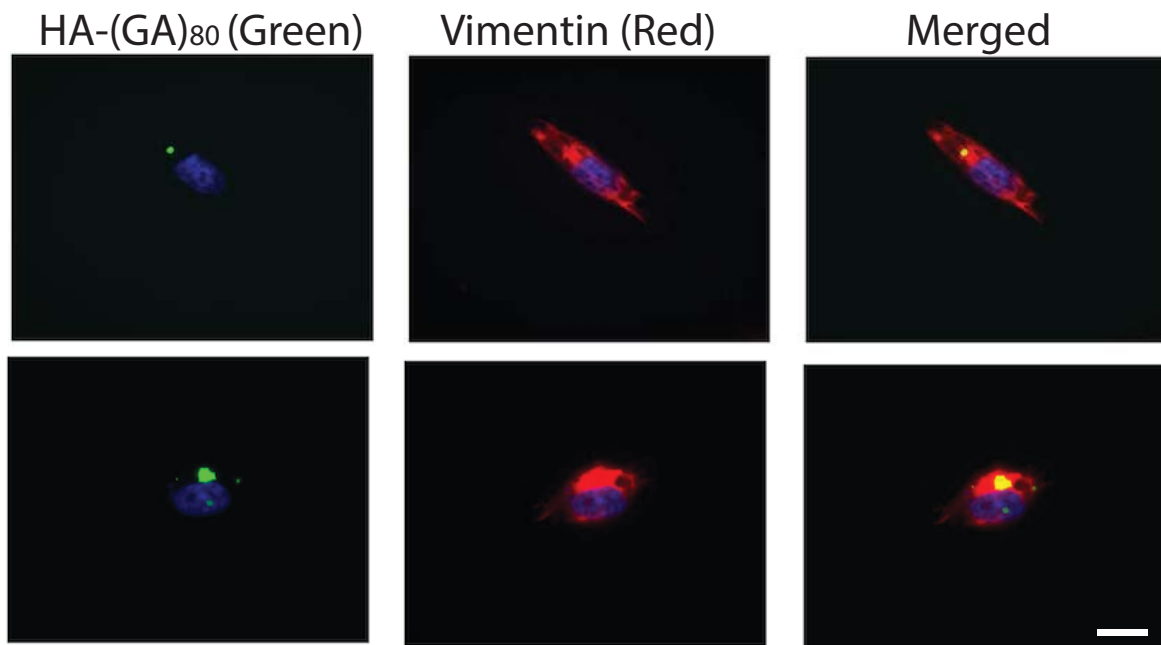

b

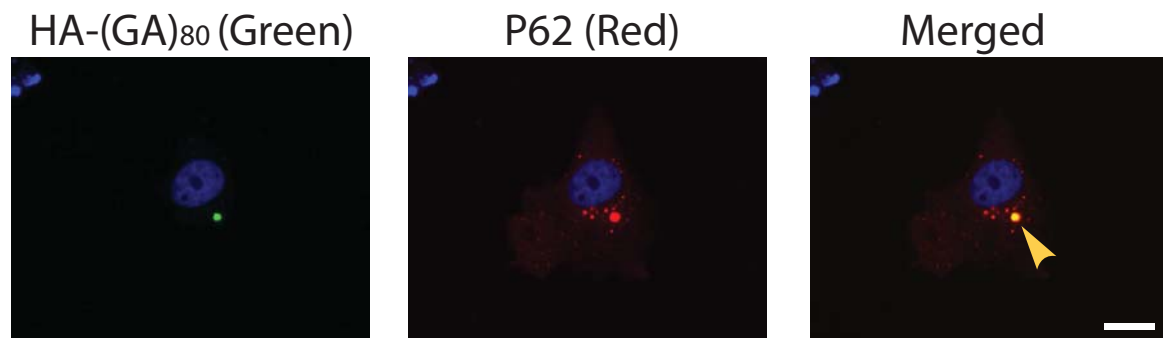

Supplement: Supplementary file 5 — Supplementary material 5 (PDF 107 kb) [file 401_2015_1448_MOESM5_ESM.pdf]
