# Supplementary material for: FTD/ALS-associated poly(GR) protein impairs the Notch pathway and is recruited by poly(GA) into cytoplasmic inclusions
Source: Acta Neuropathol. 2015 Jun 2;130(4):525–35. doi: 10.1007/s00401-015-1448-6 (PMC4575383; doi:10.1007/s00401-015-1448-6)

Yang et al., Figure S3

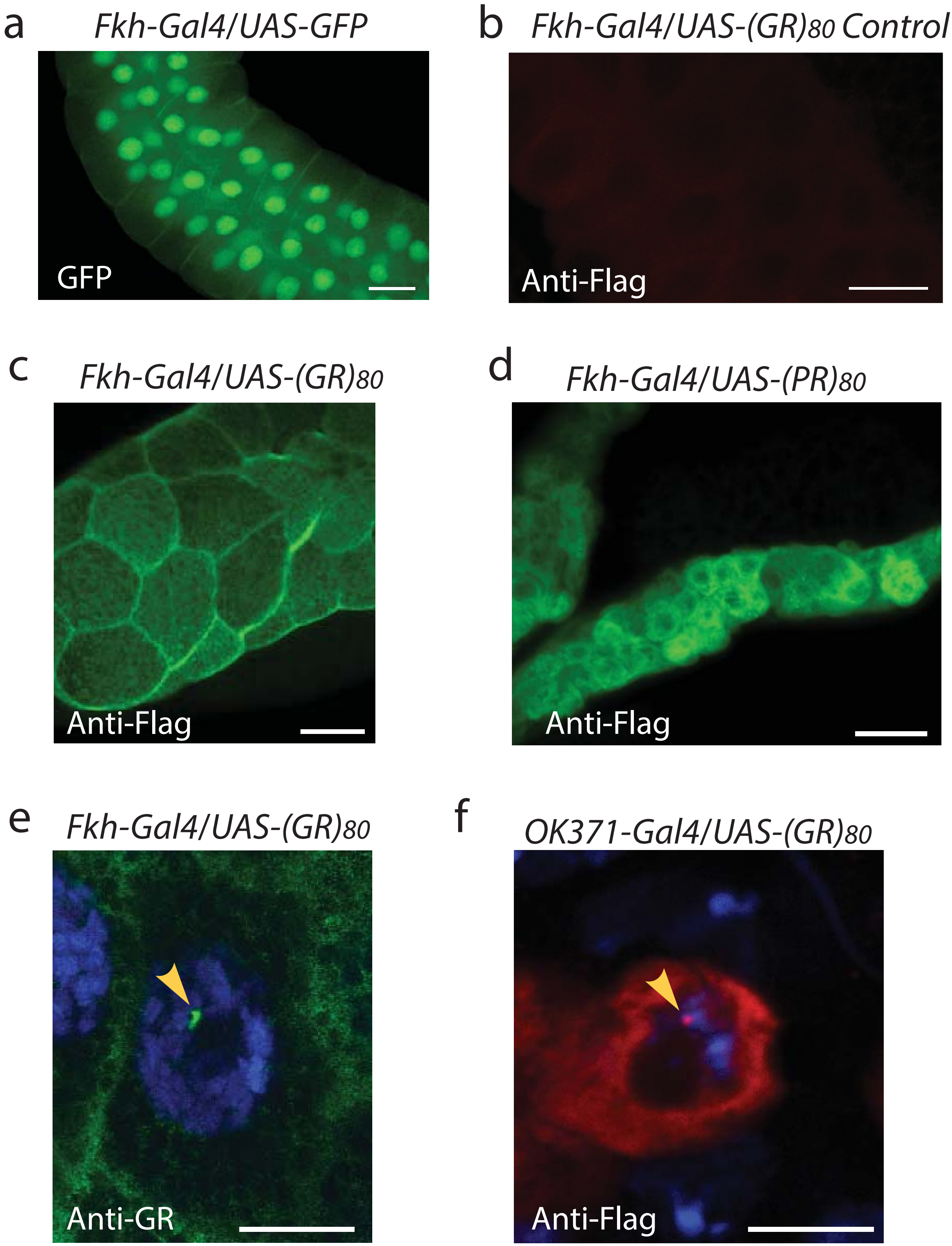

Supplement: Supplementary file 4 — Supplementary material 4 (PDF 125 kb) [file 401_2015_1448_MOESM4_ESM.pdf]
